# Selenium and hydrogen selenide: essential micronutrient and the fourth gasotransmitter?

**DOI:** 10.1186/s40635-019-0281-y

**Published:** 2019-12-16

**Authors:** Mathun Kuganesan, Kavitej Samra, Eloise Evans, Mervyn Singer, Alex Dyson

**Affiliations:** Bloomsbury Institute of Intensive Care Medicine, Division of Medicine, University College London, Gower Street, London, WC1E 6BT UK

**Keywords:** Selenium, Selenoprotein, Hydrogen sulfide, Metabolism, Redox, Oxidative stress, Oxidative phosphorylation, Cytochrome C oxidase, Mitochondria

## Abstract

Selenium (Se) is an essential micronutrient required by organisms of diverse lineage. Dietary Se is converted to hydrogen selenide either enzymatically or by endogenous antioxidant proteins. This convergent biochemical step crucially underlies the subsequent biological activity of Se and argues for inclusion of hydrogen selenide as the fourth endogenous gasotransmitter alongside nitric oxide, carbon monoxide and hydrogen sulfide.

Endogenously generated hydrogen selenide is incorporated into numerous ‘selenoprotein’ oxidoreductase enzymes, essential for maintaining redox-status homeostasis in health and disease. Direct effects of endogenous hydrogen selenide on cellular and molecular targets are currently unknown. Given exogenously, hydrogen selenide acts as a modulator of metabolism via transient inhibition of mitochondrial cytochrome C oxidase. Here we provide an overview of Se biology, its impact on several physiological systems (immune, endocrine, cardiovascular and metabolic) and its utility as a supplement in acute and critical illness states. We further explore the evidence base supporting its role as the fourth gasotransmitter and propose a strategic case towards generation of novel selenomimetic therapeutics.

## Introduction

Elemental selenium (Se; Greek [σελήνη] selene or ‘moon’) was discovered in 1817 by Swedish chemists Jöns Jakob Berzelius and Johan Gottlieb Gahn [1]. On communication of its discovery, Berzelius stated that *‘*I have just examined it [selenium] more carefully and have found that what we took for tellurium is a new substance, endowed with interesting properties. This substance has the properties of a metal, combined with that of sulfur, to such a degree that one would say it is a new kind of sulfur’ [2]*.*

**Fig. 1:**
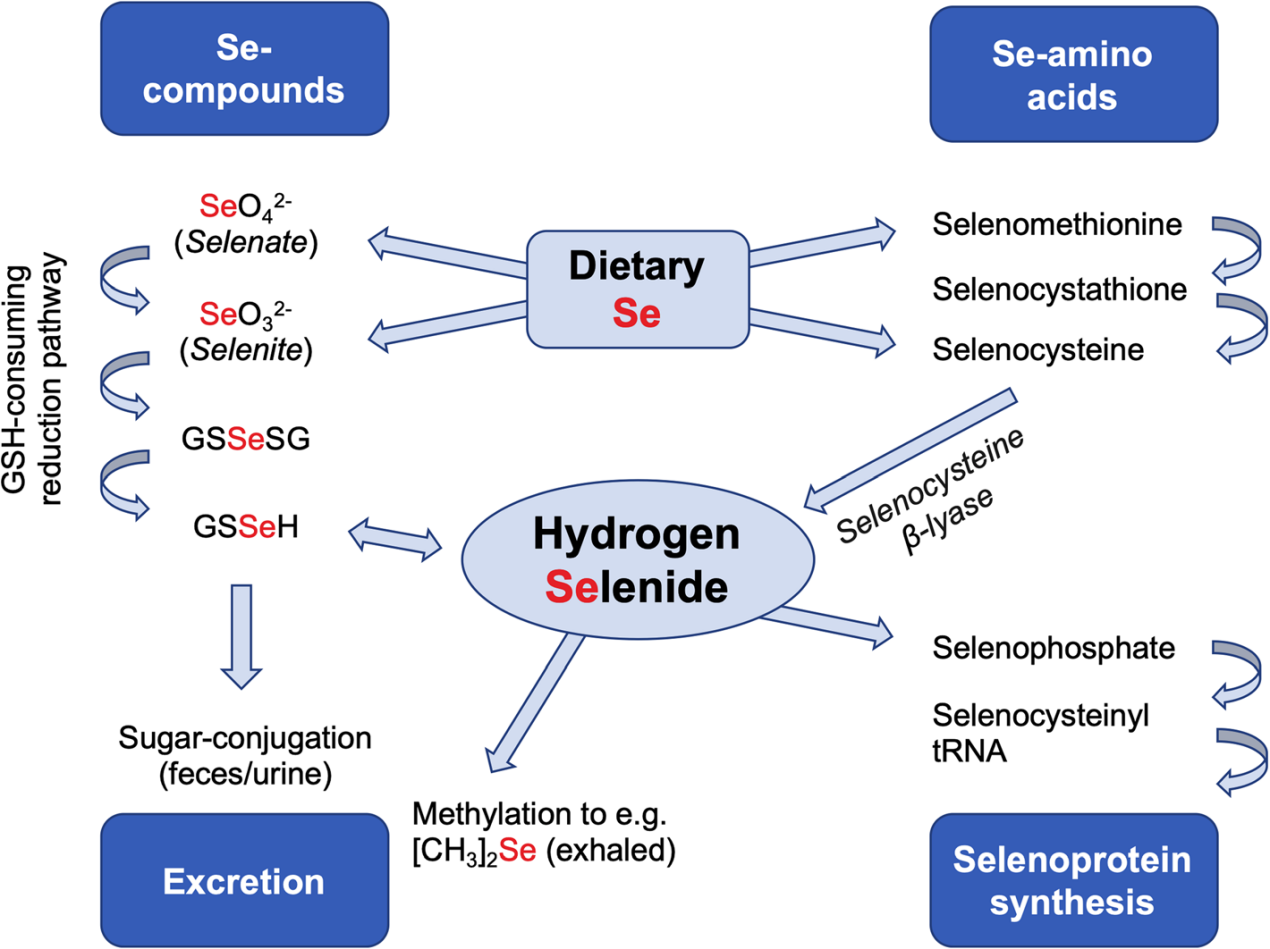
Uptake and metabolism of Se-containing compounds. [CH_3_]_2_Se; dimethyl selenide, GSH; reduced glutathione, GSSeH; glutathioselenol, GSSeSG; selenodiglutathione, tRNA; transfer ribonucleic acid

Selenium (alongside oxygen, sulfur and tellurium) belongs to the chalcogens—group 16 of the periodic table. It is an essential trace element required by organisms of diverse lineage (bacteria, archaea, eukaryotes) [3]. In addition to the elemental form, non-elemental forms and selenium-containing organic and inorganic molecules point towards a complex chemistry. Selenide is the reduced form of elemental selenium (oxidation state − 2), formed in biological systems or acidic environments from water-soluble selenium-containing compounds [4]. Endogenously generated hydrogen selenide is present as the small gaseous molecule, H_2_Se; analogous to sulfide, it is in equilibrium with the hydroselenide anion (HSe^-^) [5]. Similar to the other gaseous mediators reviewed in this issue, it also generates numerous oxidation products, the most abundant being selenite (SeO_3_^2-^) and selenate (SeO_4_^2-^), with oxidation states of + 4 and + 6, respectively [4, 6]. Crucially, conversion of all forms of intracellular Se-containing compounds to hydrogen selenide (either enzymatically or through redox reactions) represents a convergent and essential biochemical step (Fig. 1). This underpins its subsequent biological activity and, as we explore herein, adds support to its inclusion as the fourth endogenous ‘gasotransmitter’ alongside nitric oxide (NO), carbon monoxide (CO) and hydrogen sulfide (H_2_S/HS^-^).

In 2002, H_2_S was postulated to be the third member of a class of gaseous mediators, the gasotransmitters [7]. Five criteria were proposed to substantiate this claim, namely (i) they had to be small molecules of gas and (ii) freely permeable to cell membranes, (iii) they were endogenously and enzymatically generated albeit under regulatory controls, (iv) they had well-defined functions at physiologically relevant concentrations and (v) possessed cellular effects that may or may not be mediated by second messengers, but should have specific cellular and molecular targets [7]. To date, the importance of selenium in human biology has focussed on its incorporation into proteins [8, 9]. These ‘selenoproteins’, notable for their oxidoreductase activity and ability to impact on cellular redox status, have consequently drawn significant interest from the intensive care community and beyond. We propose that the physiological role(s) of endogenous hydrogen selenide extend beyond its incorporation into selenoproteins and, as a putative gasotransmitter, it is currently able to satisfy most of the above five criteria. Through a review of current knowledge and by using a hypothesis-driven approach, we here explore the evidence base arguing towards inclusion of hydrogen selenide as the fourth endogenous gasotransmitter. Necessarily, we provide an overview of selenium biology, including the functional roles of various proteins that constitute the ‘selenoproteome’. The significance of selenium and its derivatives in acute illness is considered, with a focus on immune, endocrine, circulatory and metabolic systems. Finally, we assess the utility of selenium supplementation and provide a strategic case towards the generation of new classes of putative selenomimetics.

## Endogenous generation and metabolism of hydrogen selenide

Numerous published works have referred interchangeably to elemental Se and the oxidation products of hydrogen selenide. For simplicity, we too will herein refer to the above forms as ‘Se’, unless stated otherwise. It is notable that endogenous generation and metabolism of hydrogen selenide is analogous to many aspects of its chalcogenomimetic congener, hydrogen sulfide. Elemental selenium is obtained exclusively from dietary sources [10, 11], either as the oxidation products selenite or selenate, or following incorporation of Se into the amino acids cysteine and methionine (generating selenocysteine and selenomethionine, respectively). Entry of Se into cells has been studied in vitro, whereby its accumulation intracellularly was found to be both energy-dependent or independent [12, 13]. This likely reflects uptake of either gaseous (diffusion) or anionic (active transport) forms. The seleno-amino acids are substrates for an enzymatic intracellular metabolic pathway forming endogenous hydrogen selenide (Fig 1) [14]; selenocysteine, formed from selenomethionine via transsulfuration, is converted into hydrogen selenide by selenocysteine β-lyase (SCL; EC 4.4.1.16) [15, 16]. This enzyme, first isolated in 1982 [15], catalyses decomposition of selenocysteine to hydrogen selenide and alanine, utilising pyridoxal 5’-phosphate as a cofactor [15]. SCL has been found in the human liver, kidney, heart and adrenal and muscle tissue in decreasing order of specific activity [17], and can be regulated by hypoxia, oxidative stress, pro-inflammatory cytokines and glucocorticoids [18–20].

Intracellularly, the dietary oxidation product selenate is first reduced to selenite [21] and then to hydrogen selenide, either by thioredoxin reductase [22] or through a series of redox reactions coupled to reduced glutathione (GSH) [23]. Although essential, hydrogen selenide is intrinsically toxic at inappropriately high concentrations [24]. Consequently, in excess, Se is either excreted in faeces or urine (as oxidised forms, a trimethylselenonium ion or conjugated seleno-[hexose] sugars) or, at higher concentrations, exhaled following methylation (e.g. to dimethyl selenide) [25–30].

## Selenoproteins and redox status

Selenoprotein synthesis involves incorporation of Se and this process mandates an uniquely adapted translational machinery [31, 32]. Insertion of Se at the catalytic site of selenoprotein enzymes greatly enhances their biological activity [33]. Their importance in biology is epitomised by inhibition of embryogenesis following genetic deletion of several selenoproteins [34–37]. The significance of this system has also been elegantly illustrated in vitro by showing that a reduction in selenoprotein abundance (through an antisense oligonucleotide) resulted in substantial increases in cellular reactive oxygen species (ROS) [38]. Cytochrome c- and caspase-dependent apoptosis was subsequently observed, yet incubation with the antioxidants glutathione, α-tocopherol and N-acetylcysteine, either alone or in combination, was unable to prevent cellular death [38].

This finding exemplifies the homeostasis of redox status as critical in health, and its dysregulation being a prominent factor across myriad pathologies. Many of these are encountered in critically ill patients (e.g. hypoxic or inflamed states), whereby excessive ROS production and/or inadequate detoxification generates significant oxidative stress with subsequent damage to lipids, proteins and DNA [39, 40]. Se plays an important role in maintaining redox homeostasis [41] following incorporation into the key antioxidant selenoenzymes, glutathione peroxidase (GPx), thioredoxin reductase (TrxR) and methionine sulfoxide reductase (Msr) [8]. Twenty-five selenoproteins have been characterised to date; the majority have been ascribed definitive and diverse functional roles and have been reviewed in detail elsewhere [42]. Cytosolic GPx-(isoform 1) was the first mammalian selenoprotein enzyme to be discovered [43–45]. Subsequently, a further four Se-containing isoforms have been identified (collectively, GPx; 1–4 and 6). The most abundant are GPx1, primarily involved in detoxifying intracellular hydrogen peroxide (H_2_O_2_) to water, and GPx4 with a greater affinity for lipid hydroperoxides [46]. Both prevent lipid peroxidation, thus limiting cellular damage in states of oxidative stress. TrxR consists of three isoenzymes involved in regulating the redox status of the antioxidant protein thioredoxin (Trx). Like GPx, Se-containing TrxR can catalyse reduction of H_2_O_2_ and organic hydroperoxides [47] and further functions to regulate DNA repair and intracellular redox signalling [48, 49]. Additional redox-active selenoproteins include (i) Msr, where the ratio of the reduced/oxidised form (of methionine) communicates cellular redox status [50], (ii) selenoprotein P that acts principally as a Se carrier but also undergoes redox reactions [51–54] and (iii) deiodinases that are oxidoreductase enzymes crucial for thyroid function [55].

Decreased plasma Se levels (up to 40%) are well recognised in heterogenous medical/surgical patient cohorts and ICU patients [56–58]. Of note, the level of deficiency is a prognosticator and correlates with disease severity [56–58]. Mechanism(s) underlying these deficiencies remain incompletely understood. Se has been described as a negative acute phase reactant; its deficiency could relate to either decreased Se intake or increased metabolism of Se-containing compounds. However, the most frequently cited cause is redistribution [57–60]. There is scant evidence that this deficiency, recorded in blood, translates to a reduction in selenoprotein synthesis in vital organs. However, in chronic deficiency states, a significant correlation is observed between plasma Se and erythrocyte GPx activity [61, 62]. Further sequelae of Se deficiency and its impact on several physiological systems relevant to critical illness are discussed below.

## Immunity

Se intake influences its bioavailability to the immune system; selenium supplementation in healthy volunteers increased mRNA abundance of three selenoproteins (R, S and W) in peripheral blood mononuclear cells [63]. Classic innate immunity involves macrophages playing a vital role in controlling inflammatory status and phagocytosis of pathogens [64]. Se supplementation in mice conferred a switch in macrophage phenotype from the pro-inflammatory M1 to the anti-inflammatory M2 subtype following lipopolysaccharide (LPS) exposure [65]. Selenocysteine tRNA knockout mice exhibited reduced macrophage migration [66]. The transcription factor, nuclear factor (NF)-κB, is a significant component of innate immunity and a key mediator of inflammation. A murine macrophage cell line exposed to *S. aureus* had reduced NF-kB activation following Se treatment, with decreased expression of pro-inflammatory cytokines [67]. NF-kB inhibition was also observed following selenomethionine supplementation with decreased LPS-induced inflammation in chicken trachea [68].

While these reports indicate that Se acts an anti-inflammatory micronutrient, the impact of Se supplementation on adaptive immunity has yielded more variable results. CD4^+^ T helper cells can differentiate into various subtypes depending on the nature of stimulation. A high-Se diet administered to mice promoted differentiation of naive CD4^+^ T helper cells to a (pro-inflammatory) Th1 CD4^+^ phenotype [69]. By contrast, increased IL-4 mRNA concentration, an anti-inflammatory cytokine marker for the Th2 CD4^+^ T helper cell, was observed in mice administered a low-Se diet [69]. This suggests a restricted Se diet could result in a more favourable phenotypic switch in inflamed states, however, decreased Se abundance (and hence selenoprotein synthesis) also reduced T cell maturation, a necessary step for B cell signalling [70]. More globally, a U-shaped curve has been described for the relationship between cancer prevention and blood Se concentrations [71]. A relationship was reported between mortality (all-cause, cardiovascular and cancer-related) and Se status [72]. A significant increase in mortality in patients with selenoprotein activity outside the optimal range has been suggested [72]. This implies that for immunity and beyond, achieving an ‘adequate’ Se status is more likely to be favourable over supra- or under-supplementation.

## Endocrine system

Se plays a key role in the endocrine system, particularly the thyroid gland where it is directly involved in thyroid hormone metabolism and protects against oxidative stress during thyroid hormone synthesis [73]. Adequate Se provision is necessary for the prevention of thyroid disease [74]. Indeed, relatively high concentrations of Se are present in the thyroid gland compared with other organs [75]. The most important selenoprotein classes involved in endocrine function are (i) deiodinases that convert thyroxine (T4) to the more biologically active triiodothyronine (T3), (ii) GPx that is responsible for glandular protection from ROS during generation of T4 and T3 and (iii) selenoprotein P (binding Se in plasma) that acts as a distributor to key organs (including the thyroid) when Se is restricted [74].

The importance of Se to thyroid function was demonstrated in rats administered a low-Se diet. GPx activity in the thyroid was reduced by 50% with concurrent though lesser falls in T4 and T3 synthesis [75]. This supports evidence that Se supply to deiodinases is prioritised over other selenoproteins such as GPx [74]. A fall in T4 and T3 upregulates synthesis and secretion of thyroid-stimulating hormone (TSH); this accelerates conversion of T4 to T3, generating significant quantities of hydrogen peroxide. Given the reduced activity of GPx in Se-deficient states, this can augment fibrosis of the thyroid and subsequent dysfunction [74]. Necrosis and fibrosis of the thyroid gland, particularly after iodide loading, is exacerbated by Se deficiency [76]. Given the aforementioned reduced availability of Se in critically ill patients, this could represent an important endocrine consideration for this cohort.

In addition to critically ill patients presenting with a low Se status on admission, they also typically show a combination of decreased T4 and T3 and an increase in reverse T3, with TSH within the reference range. This state is collectively known as the non-thyroidal illness syndrome (NTIS). Although considered to be protective in the first instance as an immediate, acute-phase response to nutrient restriction (and injury), NTIS is a prognosticator with likely complex implications for patients [77]. In a small cohort of trauma patients, a correlation was seen between Se and T3 deficiency, along with a parallel decrease in T4 deiodination [78]. At present, this relationship is associative rather than causal. However, given the integral function of selenoproteins in thyroid function, a direct role is plausible and warrants further investigation.

## Circulatory system

A role for Se in the cardiovascular system is particularly notable with regard to its dietary provision, a factor that shows significant geographical variation. For example, populations with Se deficiency reside in areas of low-selenium soil concentration, most notably in regions of China. By contrast, areas of Venezuela are considered ‘seleniferous’ due to high soil levels and therefore agricultural produce content [79, 80]. Keshan disease, named after a region of Heilongjiang province in China, is characterised by congestive cardiomyopathy caused by a combination of dietary Se deficiency and the presence of a mutated strain of Coxsackie virus [81]. Although it still exists in rural areas where treatment is not readily available, Se administration offers a preventative measure and is reputed to protect the infected myocardium against oxidative stress [82]. In Se-deficient individuals, a lack of GPx activity resulted in increased peroxide levels and decreased prostacyclin synthetase activity [83]. Decreased prostacyclin, increased thromboxane levels and a reduction in prostacyclin/thromboxane ratio can result in a platelet pro-aggregatory state and systemic vasoconstriction [84], potentially increasing the risk of thromboembolic events and coronary disease. An extensive literature reviewed in detail elsewhere [85] has focussed on Se deficiency contributing to the oxidation of low-density lipoproteins. These modified fats are subject to uncontrolled uptake by M2-macrophage foam cells and contribute to atherosclerotic plaques. Accordingly, numerous meta-analyses have reported on Se-status (mostly serum Se levels) and the epidemiology of cardiovascular disease [86–89]. These studies unequivocally show that a low-Se status is associated with an increased incidence of cardiovascular disease, comprising both coronary heart disease and stroke. However, as the adjusted risk between studies shows considerable variation, there is insufficient evidence to recommend Se treatment as a preventive therapy [89].

Other gasotransmitters (most notably NO and hydrogen sulfide) exert significant effects on vascular tone through well-defined cellular and molecular mechanisms [90, 91]. The administration of these molecules (or their derivatives) are antihypertensive while their inhibition (for example using nitric oxide synthase inhibitors) can increase blood pressure in states of circulatory shock. Direct vasoactive mechanisms of Se derivatives have yet to be explored, however, we postulate that they may show similar actions to the accepted members of the gasotransmitter class. Elsewhere, a role for a selenomimetic group of compounds, the phenylaminoalkyl selenides, has been reported [92]. Although their mechanism of action likely excludes a direct role for hydrogen selenide, the redox chemistry of the selenium moiety allows these molecules to propagate an ascorbate redox cycle in adrenergic nerve terminals, regulating a key enzyme of catecholamine metabolism, dopamine-β-monooxygenase. This limits conversion of dopamine to norepinephrine with resulting blood pressure–lowering effects [93].

## Metabolism

A landmark study in 2005 showed that mice breathing hydrogen sulfide gas entered a profound and reversible ‘suspended animation-like state’ [94]. Ten years later, the same group reported on the ability of exogenous hydrogen selenide gas to also induce significant metabolic effects [95]. A four-fold decrease in oxygen consumption and carbon dioxide production was observed in H_2_Se-treated mice, as well as a fall in core temperature [95]. The inhibitory metabolic role of exogenous hydrogen sulfide is however contentious as endogenous sources support basal bioenergetic function [96]. Nonetheless, when considering pharmacology alone (and a putative role for metabolism-modifying therapeutics), similarities in the effects of exogenous sulfide and selenide gases on aerobic respiration were noted; this prompted a subsequent commentary citing potential inclusion for hydrogen selenide as the fourth endogenous gasotransmitter [97].

Hydrogen selenide is likely to modulate aerobic metabolism, especially given the proximity of selenium to oxygen and sulfur in the periodic table, with both being notable for their interaction with mitochondria, and the known metabolic activity of NO, CO and hydrogen sulfide. While precise molecular mechanism(s) of metabolic inhibition in mice breathing (exogenous) H_2_Se were not elucidated [95], we propose mitochondrial cytochrome C oxidase (Complex IV) inhibition as a major pathway. We discovered in preliminary experiments using rat liver homogenate that the basic salt, sodium hydrogen selenide (NaHSe), could inhibit oxygen consumption in respiring tissue and, specifically, mitochondrial cytochrome C oxidase activity [98]. A notable difference between hydrogen selenide and hydrogen sulfide with regard to cytochrome C oxidase inhibition was the duration of effect. Hydrogen selenide acted in a transient, reversible manner, with a duration of action more than 3-fold shorter than hydrogen sulfide.

We have long held an interest in the use of mitochondrial inhibitors as adjunct therapies during revascularisation of ischemic organs. We have argued that short-term inhibitors of cytochrome C oxidase can prevent reperfusion-induced overproduction of mitochondria-derived ROS, while still allowing a degree of metabolism that supports cellular function [99, 100]. The transient nature of inhibition with hydrogen selenide could reveal it to be an attractive therapeutic target. Moreover, NaHSe given to mice at reperfusion protected against myocardial ischemia/reperfusion injury [95]. Selenide (but not the oxidation product, selenite) could target injured tissues with (radioactive) Se accumulation in the heart directly correlating to injury severity [95].

## Selenomimetics and selenium supplementation

Given the unique and intriguing biochemistry of Se, and its impact on physiology in health and disease, several selenomimetics have been trialled across diverse pathologies. The most frequently studied is ebselen, an organoselenium compound cited in over 1000 reports [101]. Ebselen catalyses the reduction of hydroperoxides by thiol compounds (e.g. glutathione), thereby mimicking the enzymatic activity of GPx [93]. In preclinical studies, it has proven efficacy in numerous models ranging from cardiovascular and neurodegenerative diseases, to alcoholic liver disease and cancer [101]. In Europe, it did not continue beyond Phase I human studies owing to toxicity concerns of the Se moiety [101]. Clinical trials in Japan however assessed the efficacy of ebselen against oxidative tissue damage following acute ischemic stroke; although showing initial promise [102], it was later discontinued due to lack of (long-term) efficacy [103]. That notwithstanding, a recent review cites a remaining appetite for the development of this molecule [104]. Other notable selenomimetics include selenazofurin, an anti-neoplastic and anti-viral agent; selenotifen, an histaminergic anti-allergic drug; and selenium sulfide, an anti-viral compound used for treating seborrhea and tinea versicolor [93].

The use of selenomimetics, either as pharmacological tools or nutritional supplements, has yet to reach fruition in acute medicine and critical care. To date, 19 clinical trials have assessed Se supplementation in critically ill patients [105–123]. A meta-analysis found a significant but modest reduction in overall mortality and length of hospital stay; however, other endpoints showed no significant difference, including 28-day all-cause mortality, length of ICU stay, incidence of new infections and duration of mechanical ventilation [124]. The cohorts analysed were heterogenous, comprising elective (cardiac) surgical patients, traumatic brain injury, sepsis and acute pancreatitis. Furthermore, the quantity of Se administered also varied considerably, both in the amount given as a loading dose (500–4000 μg), and the presence and quantity of subsequent doses or continuous infusions. This represents the prototypical conundrum of who, when and how to treat, how to assess the efficacy of the therapy and when to discontinue treatment. The likely corollary is that some patients may benefit while others may be harmed or unaffected, and the resulting meta-analysis, perhaps predictably, revealed only modest improvement or no overall effect.

The oxidation status of Se supplements could also be of importance and, to date, overlooked. The aforementioned clinical trials all utilised intravenous sodium selenite and this oxidation product would require reduction by endogenous antioxidants (e.g. glutathione, thioredoxins) to bioactive selenide. It is feasible that this may not be achievable in some critically ill patients in whom antioxidant defences are already strained. Furthermore, the aforesaid study in mice [95] found that hydrogen selenide but not selenite was effective in mitigating reperfusion injury, thus suggesting the oxidation status of the therapy does impact upon its efficacy. Additionally, lessons should be learned from initial studies using basic sulfur salts where non-targeted release of sulfide had resulting implications for pharmacokinetics, safety and efficacy. Thereafter, intelligent drug design yielded complex molecules that enabled more controlled sulfide delivery [125–127], improved targeting to its intended site of action (e.g. the mitochondrion) and at concentrations that better reflect those derived from endogenous sources [128, 129]. We postulate that the same may hold true for Se-based compounds, whereby using bespoke complex molecules that target the intended matrix, therapeutic concentrations could be delivered in a controllable manner yielding more favourable results.

## Conclusion

The indispensable micronutrient Se undergoes intracellular conversion to endogenous and bioactive hydrogen selenide. This makes a case for its inclusion as the fourth endogenous gasotransmitter alongside CO, NO and H_2_S. Hydrogen selenide currently satisfies most but not all of the criteria required for its full inclusion as a gasotransmitter. It is present physiologically as a small gaseous molecule and capable of passive transmembrane transport. It is generated enzymatically and non-enzymatically in pathways similar to hydrogen sulfide, and regulation of the enzymatic process can be modified by physical stressors and endogenous signalling molecules. Thereafter, selenoprotein synthesis is essential for the preservation of redox-balance over numerous physiological systems. Hydrogen selenide has yet to be ascribed further functional roles and is currently not associated with other well-defined cellular and molecular targets. However, we have recently demonstrated that exogenous administration can modulate aerobic respiration via inhibition of mitochondrial complex IV, and its role as an endogenous signalling molecule is under further investigation. We propose that intelligent selenomimetic drug design and delivery may generate more favorable pharmacological (and nutritional) tools over those currently used, providing an array of novel therapeutics that can confer protection against redox-based pathologies encountered in acute medicine and critical illness.

## Data Availability

Available from the corresponding author on reasonable request.

## Abbreviations

CO: Carbon monoxide
DNA: Deoxyribonucleic acid
GPx: Glutathione peroxidase
GSH: Reduced glutathione
GSSeH: Glutathioselenol
GSSeSG: Selenodiglutathione
H_2_O_2_: Hydrogen peroxide
H_2_S: Hydrogen sulfide (gas)
H_2_Se: Hydrogen selenide (gas)
HS^-^: Hydrosulfide anion
HSe^-^: Hydroselenide anion
IL: Interleukin
LDL: Low-density lipoprotein
LPS: Lipopolysaccharide
mRNA: Messenger ribonucleic acid
Msr: Methionine sulfoxide reductase
NaHSe: Sodium hydrogen selenide
NF-κB: Nuclear factor kappa B
NO: Nitric oxide
NTIS: Non-thyroidal illness syndrome
ROS: Reactive oxygen species
Se: Selenium
SCL: Selenocysteine β-lyase
SeO_3_^2-^: Selenite
SeO_4_^2-^: Selenate
T3: Triiodothyronine
T4: Thyroxine
TNF-α: Tumour necrosis factor alpha
tRNA: Transfer ribonucleic acid
Trx: Thioredoxin
TrxR: Thioredoxin reductase
TSH: Thyroid stimulating hormone
